# Study of epirubicin sustained–release chemoablation in tumor suppression and tumor microenvironment remodeling

**DOI:** 10.3389/fimmu.2022.1064047

**Published:** 2022-12-20

**Authors:** Liangliang Meng, Zhenjun Wang, Zhonghui Hou, Hufei Wang, Xiao Zhang, Xiaobo Zhang, Xiaofeng He, Xin Zhang, Boyu Qin, Jing Li, Zhongliang Zhang, Xiaodong Xue, Yingtian Wei

**Affiliations:** ^1^ Department of Radiology, Chinese People's Armed Police (PAP) Hospital of Beijing, Beijing, China; ^2^ Department of Radiology, Chinese People's Liberation Army (PLA) General Hospital, Beijing, China; ^3^ National Laboratory for Molecular Sciences, Institute of Chemistry, Chinese Academy of Sciences, Beijing, China; ^4^ Department of Oncology, Chinese People's Liberation Army (PLA) General Hospital, Beijing, China; ^5^ Department of Radiology, Characteristic Medical Center, Chinese People’s Armed Police Force, Tianjin, China

**Keywords:** chemoablation, percutaneous injection, epirubicin (EPI), drug sustained release, tumor microenvironment, immunotherapy, immune checkpoint inhibitor (ICI)

## Abstract

**Introduction:**

Although intratumoral chemoablation can obtain an impressive therapeutic effect, there is still incomplete ablation and tumor recurrence in some patients. This could be due to the short retention time of the drug in the tumor, the limited distribution of intratumoral drugs, and, beyond that, the immunotolerance caused by the tumor microenvironment (TME). There is still an urgent need to find an optimal drug sustained-release carrier and figure out the impact of regional injection to TME.

**Methods:**

In this study, we supposed to use polyethylene glycol (PEG) hydrogel as a drug carrier to improve the retention time of the drug to extend the exposure of tumor cells and investigate the feasibility of combination local Epirubicin injection with anti-PD-L1.

**Results:**

The results revealed obvious tumor suppression based on the tumor volume and the inhibition time of tumor growth in the A549 lung cancer mouse model after local injection. Furthermore, the enhanced antitumor effects of the combination of systematic anti- programmed death ligand 1 (PD-L1) therapy with local chemoablation (EPI-GEL/PD-L1) for abscopal tumor reduction in the 4T1 breast model were also observed. Flow cytometry analysis of the tumor and blood samples showed significant variations in the proportions of PD-L1^+^ and CD3^+^CD8^+^PD-1^+^ cells before and after anti-PD-L1 therapy. On day 4 after local injection of the EPI gel, the expression of PD-L1 in abscopal tumors was upregulated, while the expression of PD-L1 in bilateral tumors in mice was significantly reduced after anti-PD-L1 treatment. The proportion of CD3^+^CD8^+^PD-1^+^ cells in the tumor and circulating blood in the EPI-GEL/PD-L1 group was decreased compared with that in the EPI-GEL (single injection of epirubicin) group.

**Discussion:**

The combination of local injection of the chemoablation agent with anti-PD-L1 monoclonal antibody (mAb) therapy may strengthen the antitumor activity, and the use of PEG hydrogel as the drug carrier can extend the retention time of the chemoablation agent around the tumor, maintaining a long-term tumor-killing activity.

## Introduction

Chemical ablation is defined as the percutaneous injection of an ablation agent into a solid tumor with fine needles under imaging guidance. These agents inactivate tumor cells while remodeling the tumor microenvironment (TME) to produce a series of immunochemical reactions in order to achieve the goals of treatment; hereinafter, this is referred to as immunochemical ablation. The previously used ablation agents such as anhydrous alcohol and glacial acetic acid have been administered by local injection to rapidly dehydrate tumor cells, directly destroy cell membranes, and induce coagulation necrosis of tumor cells (1–3). However, because the speed and the scope of dispersion are not easy to control, and local pain stimulation is severe, the clinical application of these agents has been limited. To increase the traceability and stability of the ablation agent within the tumor and further enhance the antitumor cytotoxicity and remodeling of the immune TME, our team attempted to use chemotherapeutic drugs and iodized oil mixtures as chemoablation agents due to the advantage of the poor fluidity of iodized oils to prolong the contact time between the drug and the tumor and obtain preliminary efficacy verification.

**Figure 1:**
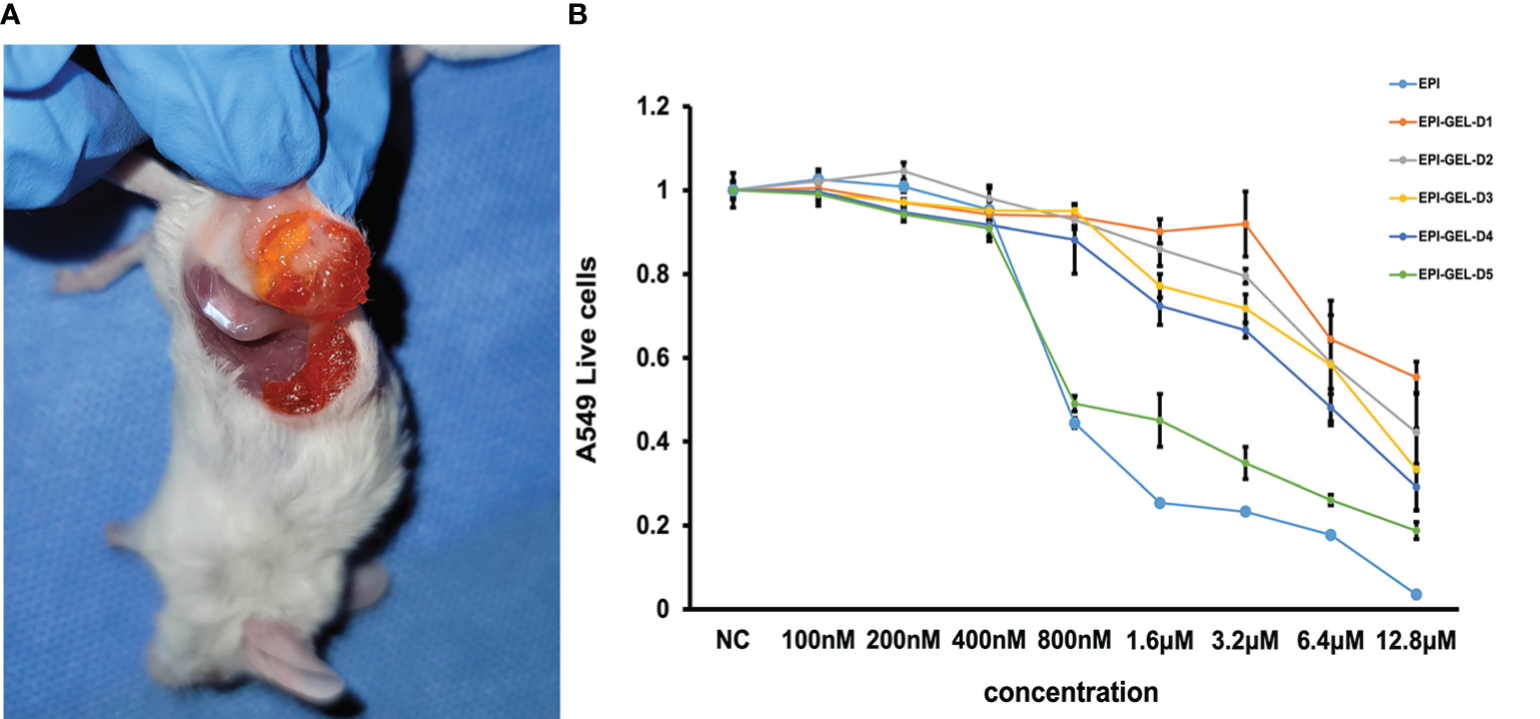
**(A)** The chemoablation agent was injected using the multipoint injection method through a dual-channel syringe to wrap the tumor with the epirubicin (EPI) gel. **(B)** EPI-loaded hydrogel sustained-release experiment showing that, with the influence of the slow release, the killing effect at the same drug concentration of EPI gel on tumor cells was not as obvious as that of the EPI solution, but the exertion of continuous inactivation of tumor cells was still evident.

Epirubicin (EPI) hydrochloride is one of the anthracycline anticancer drugs with a wide range of tumor-destroying effects and is currently a widely used ablation agent. Recent studies on anthracycline drugs have found that, apart from effectively killing tumor cells, they not only could enhance the effect of chemotherapy by stimulating the host immune system but also activate the host immune system to directly kill tumors, obtaining clinical benefits (4–10). Although intratumoral chemoablation can obtain effective tumor suppression, for some patients, there is still incomplete ablation and tumor recurrence of some parts of the tumor. The main reasons for this could be as follows: i) the degradation rate of iodide oils is slow, and the direct mixing of the EPI powder with iodized oil impedes the release of the drug; ii) it is difficult to achieve heterogeneous mixing of oil-wrapped powdered drugs; and iii) the TME causes immunotolerance to immune checkpoint inhibitors (ICIs). Therefore, in this study, we attempted to use polyethylene glycol (PEG) hydrogel as a drug carrier for tumor-wrapped injections to inhibit tumor growth through the sustained release of the drug (11, 12). In the past, the injection approach for chemoablation was intratumoral single-point or multipoint injections, through which the drug fills the entire tumor depending on the ability of the agent to spread. Due to the heterogeneity of tumors and the role of the TME, it was difficult for the intratumoral agent to spread uniformly on the entire tumor, resulting in the incomplete exposure of tumor cells to the drug and, consequently, tumor recurrence. Therefore, in this study, we intended to take advantage of the gelatinization of the hydrogel for the injection of an agent that coats the tumor completely in order to achieve sufficient exposure of the tumor to the drug (**Figure 1A**). The antitumor effect was examined by recording the tumor growth rate. In addition, as EPI induces immunogenic cell death (ICD), it may perform a key role in promoting the body’s immunogenicity. In this study, we used local chemoablation combined with systemic immunotherapy to preliminarily observe the changes in the TME from local chemoablation (13–20).

## Results

### EPI-loaded hydrogel sustained-release experiment

The *in vitro* tumoricidal activity of different concentrations of the EPI solution and EPI gel showed that the EPI gel could inactivate tumor cells through the sustained release of EPI, proving that PEG hydrogel can be used as an EPI drug carrier and does not affect the pharmacokinetics of EPI. Due to the influence of the slow-release rate, the killing effect of the same drug concentration of EPI gel on tumor cells was not as obvious as that of the EPI solution, but the exertion of a continuous inactivation of tumor cells was still observed (**Figure 1B**).

### A549 tumor-bearing mice

**Figure 2:**
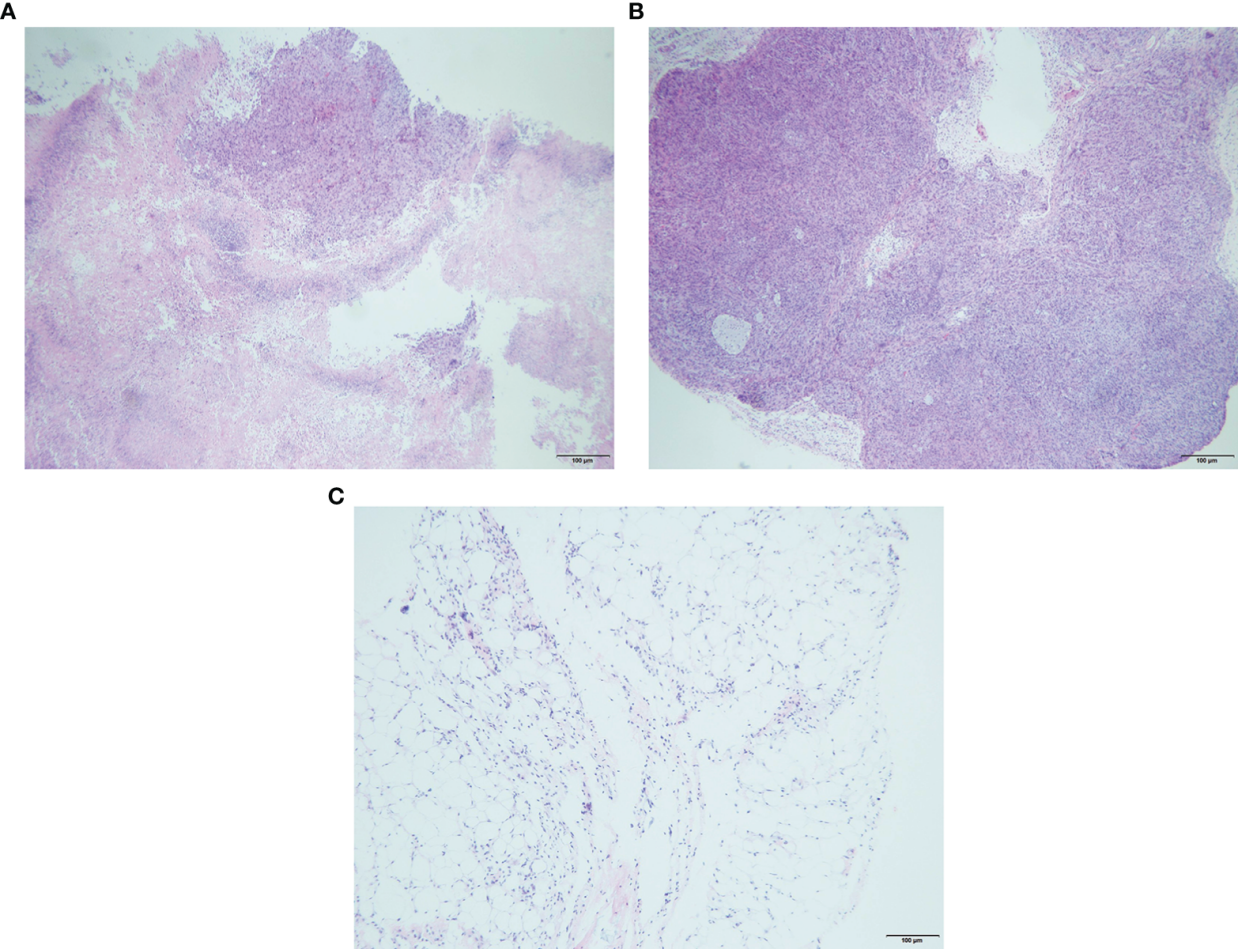
Representative hematoxylin and eosin (H&E) staining of tissue sections (right tumor of 4T1 mice) with obvious necrosis **(A)**, control tumor cells **(B)**, and peritumoral adipose tissue hyperplasia **(C)** after 3 days of epirubicin (EPI) gel injection.

**Figure 3:**
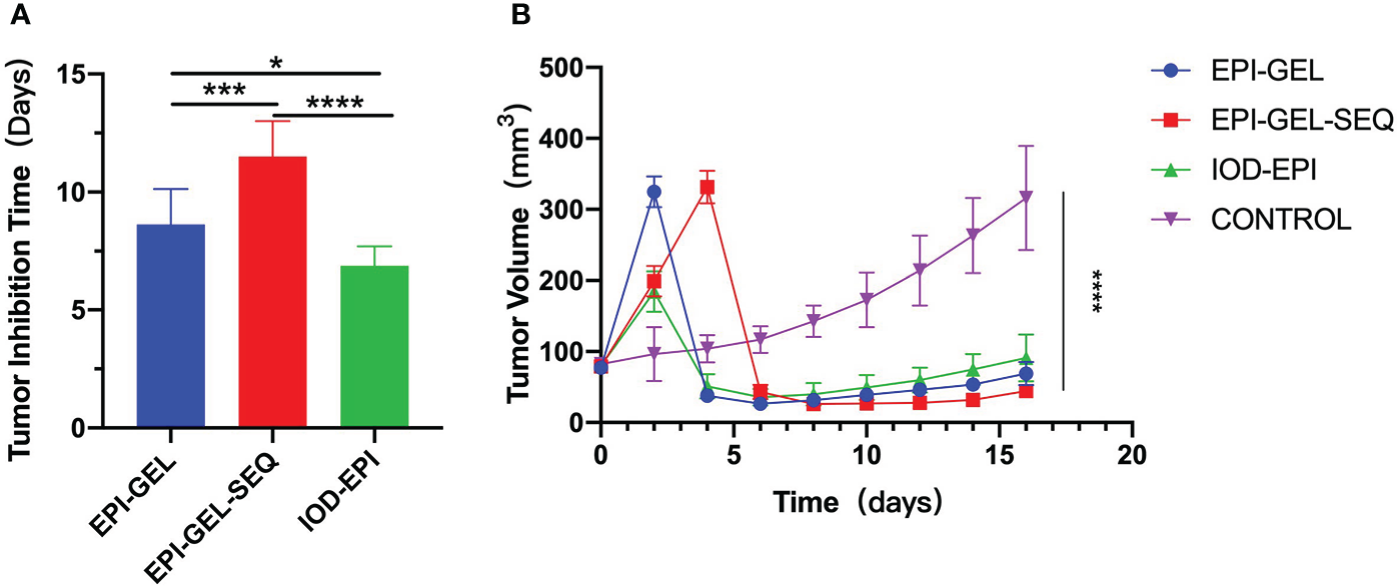
Tumor-bearing mice were monitored for tumor growth and tumor inhibition time (two successive measurements of tumor volume larger than before) after local injection. *p < 0.0332, **p < 0.0021,***p < 0.0002, ****p < 0.0001 (one-/two-way ANOVA with Tukey’s test). Data shown are the mean + SEM of the tumor inhibition time **(A)** and tumor volume **(B)**. *n* = 8/group.

**Figure 4:**
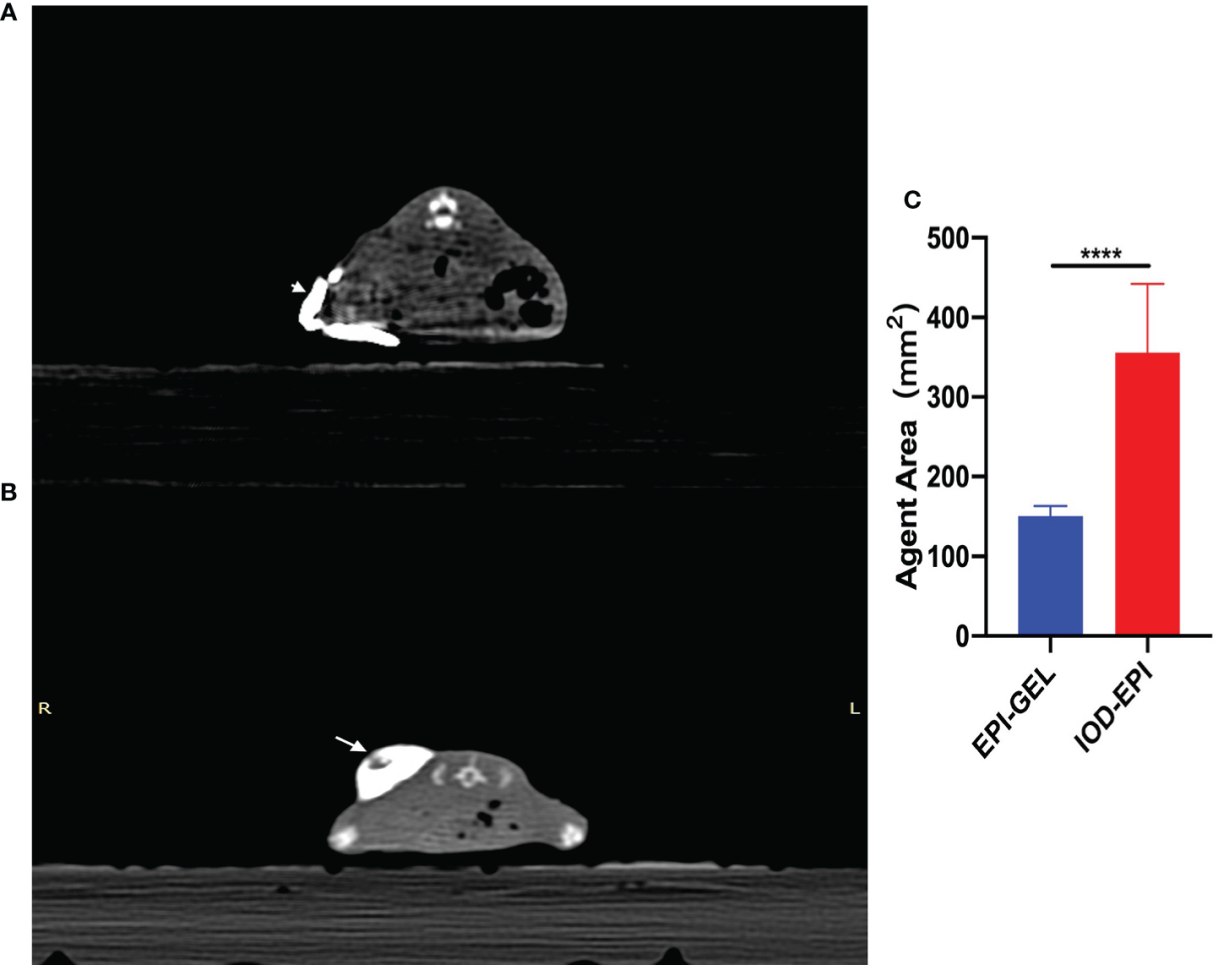
**(A, B)** Immediate post-injection CT scans showing the distribution of 400 μl epirubicin (EPI) with iodized oil as carrier (*arrowhead*) **(A)** and with polyethylene glycol (PEG) hydrogel as carrier (*arrow*) **(B)**. **(C)** The drug dispersion area (product of the maximum length and the short diameter) of the IOD-EPI (EPI powder mixed with iodinated oil) group immediately after injection was significantly larger than that of the EPI-GEL (single injection of EPI gel) group. ****p < 0.0001 (Student’s *t*-test).

Compared with the control group, after local injection of the EPI gel, the tumor grew significantly more slowly. Pathological results showed distinct necrosis visible in the tumor after EPI gel injection, and a lot of hyperplasia fibroadipose tissues can be found around the tumor (**Figures 2A–C**). The durations of inhibition of tumor growth in the group given a single injection of EPI gel (EPI-GEL group), the group administered sequential injections of EPI gel (EPI-GEL/SEQ group), and the group given EPI powder mixed with iodinated oil (IOD-EPI group) after local injection were 8.63 ± 1.5, 11.50 ± 1.5, and 6.88 ± 0.8 days, respectively (*p* < 0.0001), of which the tumor inhibition time of the sequential treatment was the most obvious and the control time of the IOD-EPI group on the tumor was shorter than that of the EPI-GEL group (**Figure 3A**). There were also differences in the inhibition of tumor volume using the three EPI injection methods, and the inhibition of tumor growth was significant when compared with the control group. The tumor volume eventually increased over time in the three groups, but the difference between the EPI-GEL and IOD-EPI groups was not significant (**Figure 3B**). CT images after local drug injection showed that the drug retention in the IOD-EPI group was worse compared with that in the EPI-GEL group (**Figures 4A, B**), and the drug dispersion area (product of the maximum length and the short diameter) of the EPI-GEL *vs*. the IOD-EPI group immediately after injection was 150.3 ± 12.6 *vs*. 355.6 ± 86.7 (*p* < 0.0001) (**Figure 4C**). It was shown that the EPI gel can maintain the peritumor drug in reserve, increase the local utilization of the drug, and reduce the toxicity of the tumoricidal drug to normal tissue.

### 4T1 tumor-bearing mice

**Figure 5:**
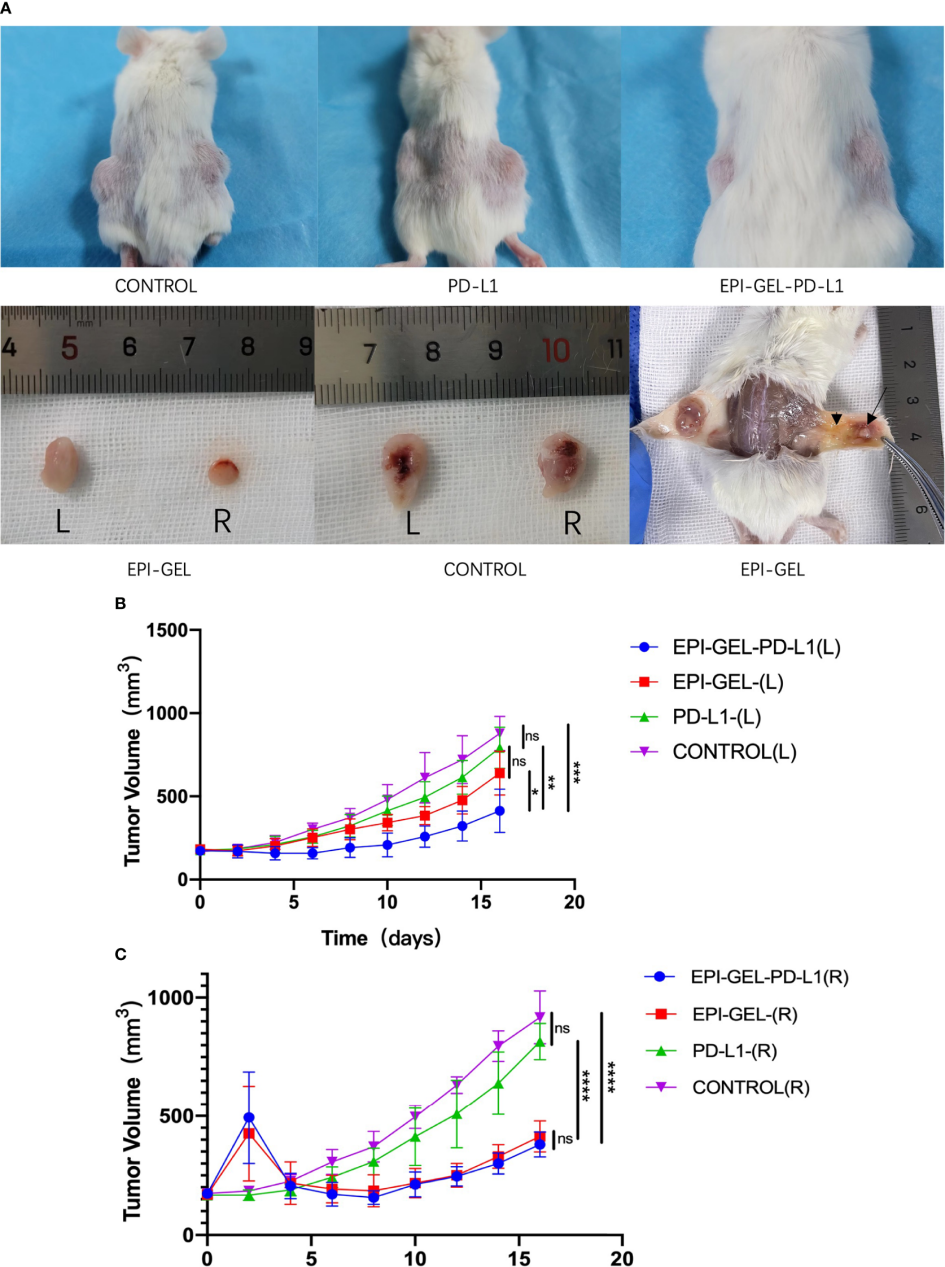
*In vivo* and *ex vivo* bilateral tumors of 4T1 mice. After injection of the epirubicin (EPI) gel, the right tumors in both the EPI-GEL (single injection of EPI gel) and EPI-GEL/PD-L1 (combined chemoablation and anti-programmed death ligand 1 therapy) groups were smaller than those in the PD-L1 and control groups. **(A)** After the degradation of the EPI gel, the right tumor shrunk (arrow) and was surrounded by inflammatory hyperplasia tissue (arrowhead). **(B, C)** Bilateral tumor growth post-injection of the EPI gel or anti-PD-L1. *ns*, not significant. *p < 0.0332, **p < 0.0021, ***p < 0.0002, ****p < 0.0001 (two-way ANOVA with Tukey’s test).

**Figure 6:**
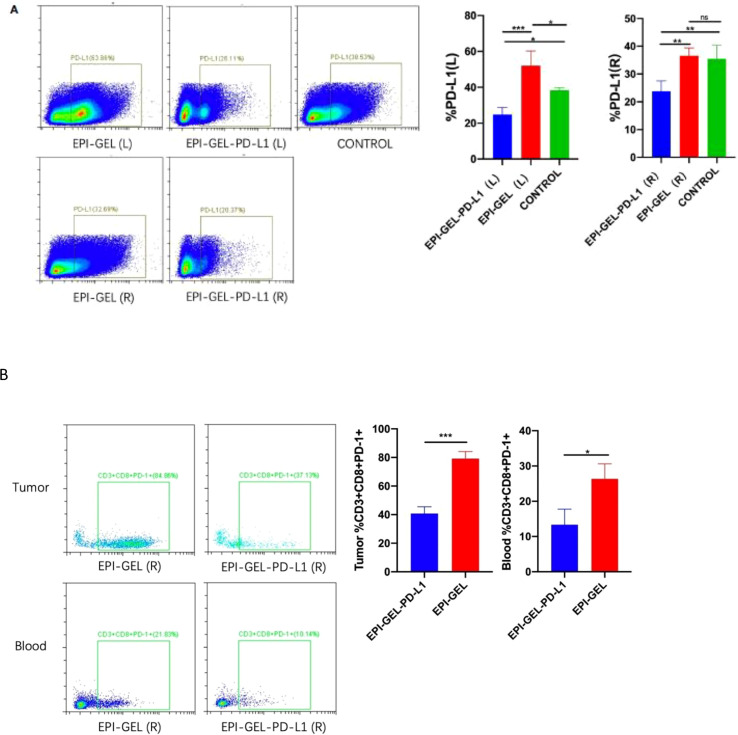
Representative flow cytometry analysis of PD-L1^+^ **(A)** and CD3^+^CD8^+^PD-1^+^ **(B)** cell infiltration in bilateral tumors and blood from mice treated with epirubicin (EPI) gel alone or in combination with anti-programmed death ligand 1 (PD-L1). ns, not significant. *p < 0.0332, **p < 0.0021, ***p < 0.0002, ****p < 0.0001 (one-way ANOVA with Tukey’s test/ unpaired t test).

Compared with the control group, the therapeutic modalities in the EPI-GEL group and the combination of systematic anti-programmed death ligand 1 (PD-L1) therapy with local chemoablation (EPI-GEL/PD-L1) led to marked tumor suppression, while the PD-L1 group did not show any difference from the control group (**Figure 5A**). None of the left tumors in the three groups were given any local interventions, but the growth of the left tumor in mice was inhibited. Over time, the growth of the tumors in the EPI-GEL and EPI-GEL/PD-L1 groups was slower than that in the control group, while the tumor growth in the EPI-GEL/PD-L1 group was slower than that in the PD-L1 group; the difference between the EPI-GEL and EPI-GEL/PD-L1 groups was significant. All of these suggest that local injection of the EPI gel into the right tumor may stimulate immunogenicity systemically (**Figure 5B**). The tumor growth in the PD-L1 group was slower than that in the control group, but there was no significant difference and no obvious tumor growth inhibition effect was shown. In the EPI-GEL and EPI-GEL/PD-L1 groups, the EPI gel was injected into the right tumor of mice. The growth of the right tumor in the mice of these two groups was significantly slower than that of mice in the control and PD-L1 groups, but there was no significant difference between the two groups. Although the tumor growth in the PD-L1 group was slower than that in the control group, no significant difference from the control group was observed (**Figure 5B**). In order to investigate the impact of local drug injection on the TME, we performed flow cytometry on tumors and the peripheral blood of mice. The results showed that 3 days after local injection of the EPI gel, the PD-L1 expression of the left tumor increased, while that in bilateral tumors in the EPI-GEL/PD-L1 group was significantly reduced (**Figure 6A**). The proportion of CD3^+^CD8^+^PD-1^+^ cells in the right tumor and circulating blood in the EPI-GEL/PD-L1 group was reduced compared with that in the EPI-GEL group (**Figure 6B**). After local injection of the EPI gel, the intratumoral proportion of CD4^+^CD25^+^Fox3^+^ cells (regulatory T cells, Tregs) in the right tumor was increased, but that in the left tumor was decreased; moreover, the CD3^+^CD8^+^ T lymphocytes in the right tumor were slightly reduced, but with no significant difference (**Supplementary Figures S1** **,** **S2**).

## Discussion

The incidence and mortality of malignant tumors are steadily increasing. The mechanisms of the TME play a significant role in drug tolerance. At the same time, the TME is also one of the important players in the resistance of tumor cells to ICIs (21–23). A low tumor mutation load, lack of tumor antigen and T-cell homing chemokine, and the immunosuppressive expression of programmed cell death 1 (PD-1)/PD-L1 will lead to immune tolerance (24, 25). In the self-growth process of solid tumors, the dense arrangement of tumor cells, the lack of a complete lymphatic system, and the malformation of supply vessels, among others, make it difficult for intravenous antitumor drugs to overcome the excessive hydrostatic pressure between tumor cells in order to reach the tumor. Combined with the influence of the extracellular matrix (ECM), certain cells, and growth factors in the TME, tumor cells build a natural “barrier” for self-protection, enhancing their resistance to chemotherapy. Although various attempts have been made by researchers, including changing the size of the drug molecules and binding to nanocarriers in order to increase the permeability of drugs in the blood vessels of tumor tissues while reducing exudation in normal tissues and the concomitant drug toxicity, the results have been less than satisfactory. Therefore, by reshaping the TME, extending the direct exposure time between tumor cells and chemotherapeutic drugs, and increasing the drug concentration, it is possible to improve the bioavailability of the drug and overcome the resistance of tumors to chemotherapeutic drugs and ICIs.

As EPI induces ICD, it has the effect of triggering the body’s immunogenicity in addition to its tumor-killing effect. Its main mechanisms of action include the following: i) it can trigger the intracellular calreticulin to move to the cell surface in order to promote antigen presentation and induce the surrounding immune cells to attack tumor cells; ii) anthracyclines stimulate the tumor cells to rapidly produce a large amount of type I interferon (IFN-1) by activating intracellular Toll-like receptor 3 (TLR3), promote the release of chemokine 10 (CXCL10), and participate in immunomodulation; and iii) anthracyclines can also induce small cell lung cancer to produce urokinase, interleukin 8 (IL-8), and monocyte chemoattractant protein 1 (MCP-1). These chemokines are the main chemical inducers of neutrophils and monocytes/macrophages (4, 26). Because of these functions, anthracyclines can induce inflammation and the interaction between immune and tumor cells, remodel the TME, and affect the biological characteristics of tumor cells in many ways. Low-dose, multi-frequency administration of anthracyclines is generally utilized to induce antitumor immunity because intravenous administration of high concentrations of drugs often causes damage to the body’s immune cells at the same time and inhibits the effect of immunotherapy. Intratumoral administration avoids damage to systemic immune cells caused by the excessive absorption of chemotherapeutic drugs into the bloodstream. A lot of studies have shown that the intratumoral injection of higher concentrations of chemotherapeutic drugs and immunoreagents is a safe and effective method (26–30). The advantages of chemoablation include the following: i) it can overcome the pressure of the tumor ECM and can percutaneously inject the drug directly into the tumor; ii) it is less traumatic with the use of fine needles to puncture the tumor percutaneously; iii) the intratumoral injection of drugs reduces the risk of systemic toxic side effects caused by the absorption of cytotoxic drugs into the blood; and iv) the flow of the ablation agent is relatively limited into the tumor, especially for lesions with capsules (27). When the agent surrounds the lesion, damage to the surrounding tissues will be avoided. Therefore, chemoablation is more suitable for tumors situated close to important organs and blood vessels and for patients with a declining performance status.

To overcome the excessive fluidity of the ablation agent, the insufficient exposure time of the tumor, and the uneven intratumoral drug distribution, we proposed using PEG hydrogel as the drug carrier for local injection. The structure of PEG hydrogel is similar to that of soft tissue, which allows nutrient diffusion, and it has good biocompatibility. Based on the reactions of the four-arm PEG amine (4-arm PEG-NH_2_) and the four-arm PEG succinimidyl succinate (4-arm PEG-SS), the four-arm PEG gel, a type of *in situ* hydrogel, is suitable for drug embedding. Its three-dimensional porous reticular structure is conducive to drug release, and, combined with its non-cytotoxicity and non-antigenicity, PEG gel is suitable for local injection as a carrier of chemoablation agents (11, 12). Previously, gels have been used by a number of researchers as a drug carrier (e.g., cisplatin or doxorubicin) for intratumoral injection and achieved expected therapeutic effects, which showed that the growth of the tumor was inhibited, but the concentration of the drug in the peritumoral tissue and bloodstream was only modest (31–34). Although a series of drug–gel delivery systems have been used in clinical trials for the treatment of head, neck, breast, and liver tumors, which indicated their effectiveness[>50% of complete and partial response (CR+PR) in head and neck region injection and >47% of objective response in recurrent or metastatic breast cancer] and safety, most of the carriers used in previous studies were thermosensitive gels (33, 35). Moreover, there are limitations to their use, including complex agent dispensation, long degradation time (>30 days or even up to 49 days as a graft), uneven distribution, are affected by ambient temperature, and the probability of reactions of the drug and reagents (31, 34). The PEG hydrogel used in this study is easy to mix, and the gelation time is not affected by external factors. In addition, compared with the previously used doxorubicin, the EPI used in this study has equal or even higher antitumor activity, but with lower toxicity and fewer side effects. Suitable gel degradation can release EPI in appropriate concentrations and cause the apoptosis of tumor cells within 48 h. It is worth mentioning that no mice died from EPI drug toxicity in this study. There are only a few investigations on a locally injectable EPI–PEG delivery system for the treatment of either lung or breast cancer, and even fewer on the combination of anti-PD-L1 treatment and EPI local injection.

The hydrogel used in this study was injected in the liquid phase through a dual-channel mixer. The mixture gelatinized in and around the tumor within 30 s, immobilized. The *in situ* gelatinization enables the drug-coated colloid to completely wrap around the tumor as much as possible, which increases the exposure time of tumor cells to the drug. This wraparound action of the ablation agent avoids the uneven distribution of intratumoral drugs due to tumor heterogeneity and may contribute to preventing tumors from invading the periphery. In this study, the hydrogel sustained-release experiment was performed to confirm that the PEG hydrogel would not affect the pharmacokinetics of EPI. The CT scan performed immediately after local injection showed that the drug was mostly immobilized in peritumor and still *in situ* after 24 h. When iodized oil is used as the drug carrier, the injection pressure diffuses the agent rapidly into the hypodermic space of the mice, and the dispersion range increases with time under the influence of the movements of mice. This may therefore limit the interaction time between the drug and the tumor cells, resulting in the waste of the drug and instead increasing the contact area between the chemotherapeutic drug and the surrounding normal tissue and, consequently, the toxic side effects.

The size of the tumors in this study was measured using a Vernier caliper. After the local injection of the EPI gel, and the swelling effect of the PEG hydrogel, the local volume of the tumor gradually increased and then shrunk with time. The injection of an ablation agent may cause local inflammation, resulting in local tissue hyperplasia and affecting the measurement of the tumor size *in vivo*. Although the bulge was always seen in the inoculation area of the mice, the macroscopic and pathological examination of the excised tumor showed reduction and necrosis. Evaluation of the treatment efficacy based on the tumor volume alone may not be accurate. Therefore, we plotted a tumor growth curve to observe the inhibition trend of the tumor growth using different treatment modalities. Both single and sequential injections of the EPI gel can inhibit tumor cell growth, with the size of the tumors being almost five times smaller than those of the control group at the end point of observation in this study. However, a single local injection of the agent may result in the incomplete inactivation of tumor cells as the EPI gel will degrade within 48 h and will cause local recurrence of the tumor over time. Sequential injections are equivalent to secondary consolidation therapy, reducing the probability of local recurrence. However, it should be noted that the concentration and the volume of the drug injected in a sequence should be able to inactivate most tumor cells; otherwise, recurrence of the tumor occurs. In individual cases, the large volume of the tumor or excessive drug leakage during subcutaneous injection will cause incomplete drug coating, resulting in insufficient tumor suppression. Therefore, we speculate that the tumor volume and the drug concentration, as well as the integrity of the looped agent, are important factors that directly impact the efficacy of tumor chemoablation. The specific correlation will be further studied in subsequent studies.

As a drug carrier, iodized oil can deliver the drug to tumor cells. But for larger tumors, its inhibitory effect is less marked than that of the EPI gel due to the extensive diffusion area of the oil-coated drug and the lower local effective utilization rate. Hence, we considered that the inhibition of tumor growth is more vulnerable to tumor size when iodized oil is used as a drug carrier. The correlation between the drug concentration, agent dose, and tumor size still needs to be determined, and specific data will be reported in our subsequent articles.

The 4T1 cell line is often used as a human stage IV breast cancer model and is inherently ineffective for immunotherapy (36). Considering the immune-boosting effect of anthracyclines, we initially attempted the combination of local EPI gel injection with anti-PD-L1 monoclonal antibody (mAb) therapy for the treatment of 4T1-bearing mice. The results showed that the local injection of the EPI gel alone and the combination of EPI gel with anti-PD-L1 therapy had significant tumor-suppressive effects, but there was no obvious difference between the two treatments. This might be related to the dose of the locally injected drug; that is, the drug dose of a single injection can have a significant tumoricidal effect, so the effect of immunotherapy in combination with the anti-PD-L1 treatment of the EPI gel-injected tumor is covered. Although the tumor growth of mice was slowed down by simple abdominal injection of the anti-PD-L1 mAb, there was no significant difference compared with the control group. There was significant inhibition of the growth of the left (non-intervention) tumors after local injection of the EPI gel and the combined EPI gel and anti-PD-L1 treatment. In addition, the flow cytometry results showed that, on day 4 after local injection of the EPI gel, the expression of PD-L1 in the left tumor was upregulated, while that in bilateral tumors in mice was significantly reduced after anti-PD-L1 treatment, suggesting that local injection of the EPI gel may have promoted immunotherapy through the exposure of more PD-L1 to the anti-PD-L1 mAb. Furthermore, we also found that the proportion of CD3^+^CD8^+^PD-1^+^ cells in the right tumor and circulating blood in the EPI-GEL/PD-L1 group was decreased compared with that in the EPI-GEL group (**Figures 5B, C**). Combined with the previously mentioned results of tumor growth inhibition in mice, we speculate that 4T1 cells may have obvious T-cell exhaustion, making them insensitive to anti-PD-L1 treatment alone; therefore, the application of anti-PD-L1 therapy alone still will not have a lasting inhibitory effect on 4T1 tumors (37, 38). When the tumor is attacked by the immune system after local EPI gel injection, the cells are regulated by the TME to improve the expression of PD-L1 (remarkable in the left tumor) as an attempt to suppress systemic immunity. However, the addition of the anti-PD-L1 agent blocks its binding pathway and improves the tumor-killing effect. As might be expected, in this study, we also observed that the proportion of CD4^+^CD25^+^Fox3^+^ cells (Tregs) in the right tumor was increased after local injection of the EPI gel, while CD3^+^CD8^+^ T lymphocytes were slightly reduced. The high concentration of a locally administered chemotherapeutic drug might destroy local immune cells in addition to killing the tumor cells, resulting in a decrease in the proportion of immune cells in the tumor environment. Therefore, combined with these preliminary results, we considered that the injection of a local chemoablation agent plays a certain role in promoting the PD-L1 expression of both *in situ* and abscopal tumors, but a part of this was transiently suppressed by the high concentration of the chemotherapeutic drug in the *in situ* tumor. Naturally, the drug leakage during the injection and the subcutaneous absorption of EPI into the blood may also affect the experimental results to a certain extent. Further research is still needed regarding the appropriate drug concentration and the frequency and timing of drug administration. Furthermore, we believe that the immune activation mechanism of the local injection of anthracyclines is not limited to upregulating the expression of PD-L1, which also needs to be further explored for the combination of pattern recognition receptor agonists such as TLR, stimulator of IFN-induced genes (STING), and retinoic-inducible gene I (RIG-I)-like receptor agonists.

## Materials and methods

### Hydrogel sustained-release experiment

To verify that the hydrogel does not affect the anticancer cytotoxicity after its binding to EPI, we conducted a sensitivity experiment of the gelatinized drugs *in vitro*. Different concentrations of EPI solutions (Pfizer Inc., New York, NY, USA) and loversol injections (Liebel-Flarsheim Canada Inc., Pointe-Claire, Canada) were proportionally mixed with 4-arm PEG-SS and 4-arm PEG-NH_2_ (Sinopeg, Xiamen, China) and then quiescently gelatinized (EPI gel; dissolved within 40–48 h). A549 and 4T1 cells were inoculated in 48- to 96-well plates, 60,000 per well for A549 and 80,000 per well for 4T1. The next day, EPI was added into the wells according to content gradients of 100, 200, 400, and 800 nM and 1.6, 3.2, 6.4, and 12.8 μM. The above steps were repeated and then 0.01 g EPI gel was added (with EPI contents of 100, 200, 400, and 800 nM and 1.6, 3.2, 6.4, and 12.8 μM) to the wells. After the addition of the EPI gel, three wells of cells were taken per concentration per day to examine the cell titers and record the cell activity values.

### Animal experiments

All experiments were approved by the Animal Ethics Committee of the Chinese PLA General Hospital. The tumor cells and BALB/c and NVSG mice were purchased from Beijing Viewsolid Biotechnology Co., Ltd. (Beijing, China).

The 4T1 and A549 tumor cells were cultured *in vitro* in 1640 RPMI medium supplemented with 10% fetal bovine serum (FBS) and 1% penicillin–streptomycin at 37°C. Cells were collected for tumor studies after reaching exponential growth during the week of culture. Subsequently, 4T1 and A549 tumor cells (2 × 10^5^) were subcutaneously injected into the bilateral flank of BALB/c mice and the right flank of NVSG mice. Female mice were used at 8–12 weeks of age and housed under specific pathogen-free conditions. When tumor volumes of 70–100 mm^3^ (A549) and 140–200 mm^3^ (4T1) were reached, the mice were randomized into different treatment groups depending on the experiment.

### A549-bearing mice

The mice were anesthetized by intraperitoneal injection of 1% sodium pentobarbital solution (60–80 mg/kg); consequently, baseline CT scans were performed. EPI was made into a solution at a concentration of 1 mg/ml. In the hydrogel group, 500 μl of the EPI solution with 4-arm PEG-SS, 200 μl of iohexol, and 300 μl of sterilized water with 4-arm PEG-NH_2_ were mixed for injection. The tumor was completely wrapped by multipoint injection using a dual-channel syringe (**Figure 1A**) of a single injection of 400 μl (200 μg EPI) (EPI-GEL group) or sequential injections of 200 μl every 3 days (EPI-GEL-SEQ group) (a total of two injections); the drug diffusion was observed by performing CT scans immediately after. In the iodinated oil group (IOD-EPI group), the EPI powder (containing 10 mg of EPI) was divided into 10 equal parts by weight. One part was mixed with 2 ml of iodinated oil (Guerbet, Villepinte, France) and stirred at uniform speed for 60 s. Thereafter, 400 μl of the agent was injected using a 1-ml syringe by multipoint injection, and the diffusion area was recorded in the CT scans. The control group did not undergo any treatment. From day 0 to day 16, the tumor size was measured daily and CT scans were performed every 3 days.

### 4T1-bearing mice

The chemoablation agent was prepared in the same way as the A549 group. In the EPI-GEL/PD-L1 group, 400 μl (200 μg EPI) of the EPI agent was unilaterally injected into the tumor (right) through a dual-channel syringe, without intervention on the left side. Thereafter, an intraperitoneal injection of 200 μl of the anti-PD-L1 mAb (10F.9G2; Bio X Cell, Lebanon, NH, USA) every 4 days was started on the second day after EPI gel injection, for a total of four injections. The EPI-GEL group received a single injection of 400 μl (200 μg EPI) of EPI gel injected into the tumor (right side) without intervention on the left side. The PD-L1 group received anti-PD-L1 antibody injected intraperitoneally every 4 days for a total of four injections. The control group did not undergo any treatment. From day 0 to day 16, the bilateral tumor size was measured daily and CT scans were performed every 3 days.

### Flow cytometry

The following mAbs and reagents were obtained from BioLegend (San Diego, CA, USA) and BD Biosciences (Franklin Lakes, NJ, USA): PE anti-mouse CD274 (B7-H1, PD-L1), APC anti-mouse CD25, PerCP/Cyanine5.5 anti-mouse CD3, FITC anti-mouse CD4, APC/Cyanine7 anti-mouse CD8a, and Brilliant Violet 421™ anti-mouse FOXP3.

On day 4 after the local injection of the EPI gel, the mice were sacrificed and the tumor and circulating blood were taken for flow cytometry analysis. The tumor was mixed with 3 ml of PBS and gently ground with a grinding rod until the tissue was broken down. Subsequently, the mixture was filtered through a 200-mesh strainer into a clean tube, centrifuged for 5 min (500 × *g*), and the supernatant discarded for later use. Then, 2 ml of erythrocyte lysate was added to the mouse peripheral blood sample. After 10 min of incubation, the sample was taken out for centrifugation for 5 min and then the supernatant was removed. Of each of the above antibodies, 2 μl was added into the tubes and the reagents were mixed at room temperature and incubated for 20 min away from light. Subsequently, 2 ml of PBS was added into the tubes, centrifuged for 5 min (500 × *g*), and the supernatant was removed. Finally, 400 μl of PBS was added into each tube for flow cytometry analysis (ACEA NovoCyte, San Diego, CA, USA).

## Data availability statement

The original contributions presented in the study are included in the article/**Supplementary Material**. Further inquiries can be directed to the corresponding author.

## Ethics statement

The animal study was reviewed and approved by The Animal Ethics Committee of the Chinese PLA General Hospital.

## Author contributions

YW designed the experiment. YW, LM, ZW, and ZH performed the experiments and wrote the manuscript. HW, XBZ, XFH, XiaZ and XinZ assisted with the experiments. BQ and JL checked the English grammar and polished the English language in the manuscript. ZZ, XX, and HW participated in the discussion. YW is the guarantor of this work and takes responsibility for the contents of the article. All authors have contributed significantly and are in agreement with the content of the manuscript. All authors contributed to the article and approved the submitted version.

## Supplementary material

Supplementary Figure 1 Representative flow cytometry analysis of CD4^+^CD25^+^Foxp3^+^ cell infiltration in bilateral tumors of mice treated with epirubicin (EPI) gel. ns, not significant. (one-way ANOVA with Tukey’s test).

Supplementary Figure 2 Representative flow cytometry analysis of CD3^+^CD8^+^ cell infiltration in the right tumor of mice treated with epirubicin (EPI) gel. ns, not significant. (Unpaired t test).

## References

[1] Rehman J Landman J Sundaram C Clayman RV. Tissue chemoablation. J Endourol (2003) 17(8):647–57. doi: 10.1089/089277903322518662 14622485

[2] Liu SR Xiao YY Le Pivert PJ Wu B Zhang X Ma XY. CT-guided percutaneous chemoablation using an ethanol-ethiodol-doxorubicin emulsion for the treatment of metastatic lymph node carcinoma: a comparative study. Technol Cancer Res Treat (2013) 12(2):165–72. doi: 10.7785/tcrt.2012.500254 22905808

[3] Ditrolio J Patel P Watson RA Irwin RJ. Chemo-ablation of the prostate with dehydrated alcohol for the treatment of prostatic obstruction. J Urol (2002) 167(5):2100–3. doi: 10.1016/S0022-5347(05)65094-X 11956449

[4] Zhang Z Yu X Wang Z Wu P Huang J. Anthracyclines potentiate anti-tumor immunity: A new opportunity for chemoimmunotherapy. Cancer Lett (2015) 369(2):331–5. doi: 10.1016/j.canlet.2015.10.002 26454214

[5] Pfirschke C Engblom C Rickelt S Cortez-Retamozo V Garris C Pucci F. Immunogenic chemotherapy sensitizes tumors to checkpoint blockade therapy. Immunity (2016) 44(2):343–54. doi: 10.1016/j.immuni.2015.11.024 PMC4758865 26872698

[6] Dosset M Vargas TR Lagrange A Boidot R Végran F Roussey A. PD-1/PD-L1 pathway: an adaptive immune resistance mechanism to immunogenic chemotherapy in colorectal cancer. Oncoimmunology (2018) 7(6):e1433981. doi: 10.1080/2162402X.2018.1433981 29872568 PMC5980491

[7] Cui S. Immunogenic chemotherapy sensitizes renal cancer to immune checkpoint blockade therapy in preclinical models. Med Sci Monit (2017) 23:3360–6. doi: 10.12659/MSM.902426 PMC5516681 28697172

[8] Steele TA. Chemotherapy-induced immunosuppression and reconstitution of immune function. Leuk Res (2002) 26(4):411–4. doi: 10.1016/S0145-2126(01)00138-2 11839388

[9] Galluzzi L Senovilla L Zitvogel L Kroemer G. The secret ally: immunostimulation by anticancer drugs. Nat Rev Drug Discov (2012) 11(3):215–33. doi: 10.1038/nrd3626 22301798

[10] Alizadeh D Larmonier N. Chemotherapeutic targeting of cancer-induced immunosuppressive cells. Cancer Res (2014) 74(10):2663–8. doi: 10.1158/0008-5472.CAN-14-0301 PMC4041515 24778417

[11] Krutkramelis K Xia B Oakey J. Monodisperse polyethylene glycol diacrylate hydrogel microsphere formation by oxygen-controlled photopolymerization in a microfluidic device. Lab Chip (2016) 16(8):1457–65. doi: 10.1039/C6LC00254D PMC4829474 26987384

[12] Alexander A Ajazuddin Khan J Saraf S Saraf S. Polyethylene glycol (PEG)-Poly(N-isopropylacrylamide) (PNIPAAm) based thermosensitive injectable hydrogels for biomedical applications. Eur J Pharm Biopharm (2014) 88(3):575–85. doi: 10.1016/j.ejpb.2014.07.005 25092423

[13] Yang Y. Cancer immunotherapy: harnessing the immune system to battle cancer. J Clin Invest (2015) 125(9):3335–7. doi: 10.1172/JCI83871 PMC4588312 26325031

[14] Schreiber RD Old LJ Smyth MJ. Cancer immunoediting: integrating immunity's roles in cancer suppression and promotion. Science (2011) 331(6024):1565–70. doi: 10.1126/science.1203486 21436444

[15] Gajewski TF Schreiber H Fu YX. Innate and adaptive immune cells in the tumor microenvironment. Nat Immunol (2013) 14(10):1014–22. doi: 10.1038/ni.2703 PMC4118725 24048123

[16] de Graaf JF de Vor L Fouchier RAM van den Hoogen BG. Armed oncolytic viruses: A kick-start for anti-tumor immunity. Cytokine Growth Factor Rev (2018) 41:28–39. doi: 10.1016/j.cytogfr.2018.03.006 29576283 PMC7108398

[17] Vitale I Shema E Loi S Galluzzi L. Intratumoral heterogeneity in cancer progression and response to immunotherapy. Nat Med (2021) 27(2):212–24. doi: 10.1038/s41591-021-01233-9 33574607

[18] Costa A Kieffer Y Scholer-Dahirel A Pelon F Bourachot B Cardon M. Fibroblast heterogeneity and immunosuppressive environment in human breast cancer. Cancer Cell (2018) 33(3):463–79.e10. doi: 10.1016/j.ccell.2018.01.011 29455927

[19] Sokratous G Polyzoidis S Ashkan K. Immune infiltration of tumor microenvironment following immunotherapy for glioblastoma multiforme. Hum Vaccin Immunother (2017) 13(11):2575–82. doi: 10.1080/21645515.2017.1303582 PMC5703406 28362548

[20] Suzuki K Kadota K Sima CS Nitadori J Rusch VW Travis WD. Clinical impact of immune microenvironment in stage I lung adenocarcinoma: tumor interleukin-12 receptor β2 (IL-12Rβ2), IL-7R, and stromal FoxP3/CD3 ratio are independent predictors of recurrence. J Clin Oncol (2013) 31(4):490–8. doi: 10.1200/JCO.2012.45.2052 PMC3731922 23269987

[21] Sharma P Hu-Lieskovan S Wargo JA Ribas A. Primary, adaptive, and acquired resistance to cancer immunotherapy. Cell (2017) 168(4):707–23. doi: 10.1016/j.cell.2017.01.017 PMC5391692 28187290

[22] Pardoll DM. The blockade of immune checkpoints in cancer immunotherapy. Nat Rev Cance (2012) 12(4):252–64. doi: 10.1038/nrc3239 PMC4856023 22437870

[23] Inoue H Tani K. Multimodal immunogenic cancer cell death as a consequence of anticancer cytotoxic treatments. Cell Death Differ (2014) 21(1):39–49. doi: 10.1038/cdd.2013.84 23832118 PMC3857623

[24] Ascierto PA. Immunotherapies and novel combinations: the focus of advances in the treatment of melanoma. Cancer Immunol Immunother (2015) 64(3):271–4. doi: 10.1007/s00262-014-1647-3 PMC11029533 25549844

[25] Hamanishi J Mandai M Ikeda T Minami M Kawaguchi A Murayama T. Safety and antitumor activity of anti-PD-1 antibody, nivolumab, in patients with platinum-resistant ovarian cancer. J Clin Oncol (2015) 33(34):4015–22. doi: 10.1200/JCO.2015.62.3397 26351349

[26] Wu J Waxman DJ. Immunogenic chemotherapy: Dose and schedule dependence and combination with immunotherapy. Cancer Lett (2018) 419:210–21. doi: 10.1016/j.canlet.2018.01.050 PMC5818299 29414305

[27] Baniel CC Sumiec EG Hank JA Bates AM Erbe AK Pieper AA. Intratumoral injection reduces toxicity and antibody-mediated neutralization of immunocytokine in a mouse melanoma model. J Immunother Cancer (2020) 8(2). doi: 10.1136/jitc-2020-001262 PMC7594540 33115944

[28] Wu X He C Wu Y Chen X. Synergistic therapeutic effects of schiff's base cross-linked injectable hydrogels for local co-delivery of metformin and 5-fluorouracil in a mouse colon carcinoma model. Biomaterials (2016) 75:148–62. doi: 10.1016/j.biomaterials.2015.10.016 26497429

[29] Brachi G Ruiz-Ramírez J Dogra P Wang Z Cristini V Ciardelli G. Intratumoral injection of hydrogel-embedded nanoparticles enhances retention in glioblastoma. Nanoscale (2020) 12(46):23838–50. doi: 10.1039/D0NR05053A PMC8062960 33237080

[30] Alvarez M Molina C De Andrea CE Fernandez-Sendin M Villalba M Gonzalez-Gomariz J. Intratumoral co-injection of the poly I:C-derivative BO-112 and a STING agonist synergize to achieve local and distant anti-tumor efficacy. J Immunother Cancer (2021) 9(11). doi: 10.1136/jitc-2021-002953 PMC8627419 34824158

[31] Al-Abd AM Hong KY Song SC Kuh HJ. Pharmacokinetics of doxorubicin after intratumoral injection using a thermosensitive hydrogel in tumor-bearing mice. J Control Release (2010) 142(1):101–7. doi: 10.1016/j.jconrel.2009.10.003 19819274

[32] Wu W Chen H Shan F Zhou J Sun X Zhang L. A novel doxorubicin-loaded *in situ* forming gel based high concentration of phospholipid for intratumoral drug delivery. Mol Pharm (2014) 11(10):3378–85. doi: 10.1021/mp500019p 24735404

[33] Burris HA 3rd Vogel CL Castro D Mishra L Schwarz M Spencer S. Intratumoral cisplatin/epinephrine-injectable gel as a palliative treatment for accessible solid tumors: a multicenter pilot study. Otolaryngol Head Neck Surg (1998) 118(4):496–503. doi: 10.1177/019459989811800412 9560102

[34] Kang GD Cheon SH Song SC. Controlled release of doxorubicin from thermosensitive poly(organophosphazene) hydrogels. Int J Pharm (2006) 319(1-2):29–36. doi: 10.1016/j.ijpharm.2006.03.032 16677786

[35] Malhotra H Plosker GL. Cisplatin/epinephrine injectable gel. Drugs Aging (2001) 18(10):787–93. doi: 10.2165/00002512-200118100-00007 11735625

[36] Yang L Yong L Zhu X Feng Y Fu Y Kong D. Disease progression model of 4T1 metastatic breast cancer. J Pharmacokinet Pharmacodyn (2020) 47(1):105–16. doi: 10.1007/s10928-020-09673-5 31970615

[37] Wherry EJ. T Cell exhaustion. Nat Immunol (2011) 12(6):492–9. doi: 10.1038/ni.2035 21739672

[38] Dolina JS Van Braeckel-Budimir N Thomas GD Salek-Ardakani S. CD8(+) T cell exhaustion in cancer. Front Immunol (2021) 12:715234. doi: 10.3389/fimmu.2021.715234 34354714 PMC8330547

