# Correction: Study of epirubicin sustained–release chemoablation in tumor suppression and tumor microenvironment remodeling

**DOI:** 10.3389/fimmu.2025.1719195

**Published:** 2025-10-13

**Authors:** Liangliang Meng, Zhenjun Wang, Zhonghui Hou, Hufei Wang, Xiao Zhang, Xiaobo Zhang, Xiaofeng He, Xin Zhang, Boyu Qin, Jing Li, Zhongliang Zhang, Xiaodong Xue, Yingtian Wei

**Affiliations:** ^1^ Department of Radiology, Chinese People’s Armed Police (PAP) Hospital of Beijing, Beijing, China; ^2^ Department of Radiology, Chinese People’s Liberation Army (PLA) General Hospital, Beijing, China; ^3^ National Laboratory for Molecular Sciences, Institute of Chemistry, Chinese Academy of Sciences, Beijing, China; ^4^ Department of Oncology, Chinese People’s Liberation Army (PLA) General Hospital, Beijing, China; ^5^ Department of Radiology, Characteristic Medical Center, Chinese People’s Armed Police Force, Tianjin, China

**Keywords:** chemoablation, percutaneous injection, epirubicin (EPI), drug sustained release, tumor microenvironment, immunotherapy, immune checkpoint inhibitor (ICI)

In the published article, there was an error in the author list, the superscripts after author Zhonghui Hou, Xiao Zhang, Xiaobo Zhang, Xiaofeng He, Xin Zhang, Zhongliang Zhang, Xiaodong Xue and Yingtian Wei (corresponding author)were wrong. The corrected author list appears below.

“Liangliang Meng ^1†^, Zhenjun Wang ^2†^, Zhonghui Hou ^2†^, Hufei Wang ^3^, Xiao Zhang ^2^, Xiaobo Zhang ^2^, Xiaofeng He ^2^, Xin Zhang ^2^, Boyu Qin ^4^, Jing Li ^5^, Zhongliang Zhang ^2^, Xiaodong Xue ^2^ and Yingtian Wei ^2^*”.

In the published article, there was an error in **Figure 6B** as published. Two figures of group EPI-GEL and group EPI-GEL-PD-L1 were swapped. The corrected **Figure 6B** and its caption “Representative flow cytometry analysis of PD-L1^+^ (A) and CD3^+^CD8^+^PD-1^+^ (B) cell infiltration in bilateral tumors and blood from mice treated with epirubicin (EPI) gel alone or in combination with anti-programmed death ligand 1 (PD-L1). ns, not significant. *p < 0.0332, **p < 0.0021,***p < 0.0002, ****p < 0.0001 (one-way ANOVA with Tukey’s test/unpaired t test)” appear below.

**Figure 6:**
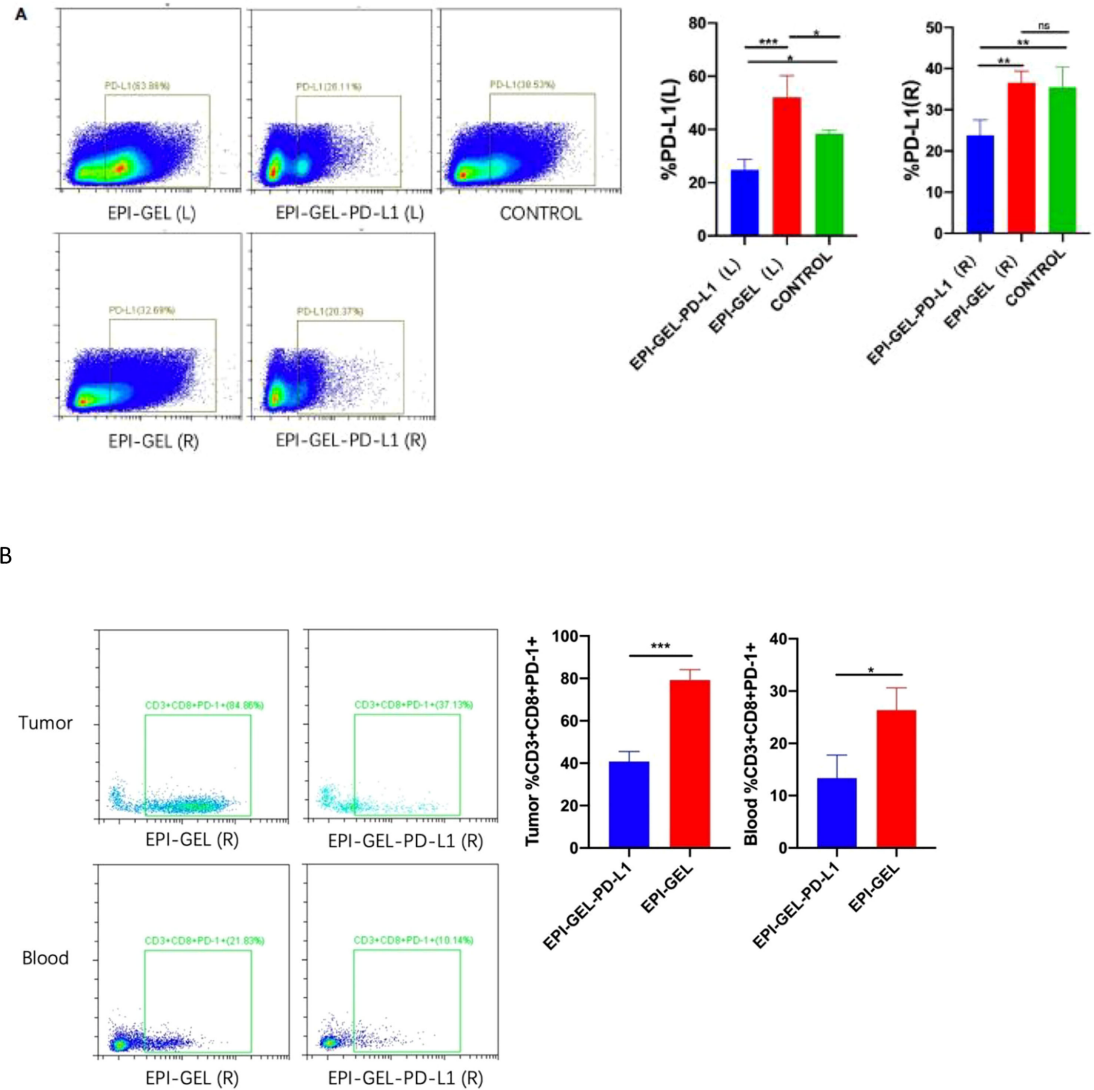
Representative flow cytometry analysis of PD-L1^+^ **(A)** and CD3^+^CD8^+^PD-1^+^ **(B)** cell infiltration in bilateral tumors and blood from mice treated with epirubicin (EPI) gel alone or in combination with anti-programmed death ligand 1 (PD-L1). ns, not significant. *p < 0.0332, **p < 0.0021,***p < 0.0002, ****p < 0.0001 (one-way ANOVA with Tukey’s test/ unpaired t test).

In the published article, there was an error. The world “expected” was incorrectly written as “expired”.

A correction has been made to **Discussion**, Paragraph 3. This sentence previously stated:

“Previously, gels have been used by a number of researchers as a drug carrier (e.g., cisplatin or doxorubicin) for intratumoral injection and achieved expired therapeutic effects, which showed that the growth of the tumor was inhibited, but the concentration of the drug in the peritumoral tissue and bloodstream was only modest (31–34).”

The corrected sentence appears below:

“Previously, gels have been used by a number of researchers as a drug carrier (e.g., cisplatin or doxorubicin) for intratumoral injection and achieved expected therapeutic effects, which showed that the growth of the tumor was inhibited, but the concentration of the drug in the peritumoral tissue and bloodstream was only modest (31–34).”

The original article has been updated.

