# Supplementary figures and images for: Study of epirubicin sustained–release chemoablation in tumor suppression and tumor microenvironment remodeling
Source: Front Immunol. 2022 Dec 20;13:1064047. doi: 10.3389/fimmu.2022.1064047 (PMC9807901; doi:10.3389/fimmu.2022.1064047)

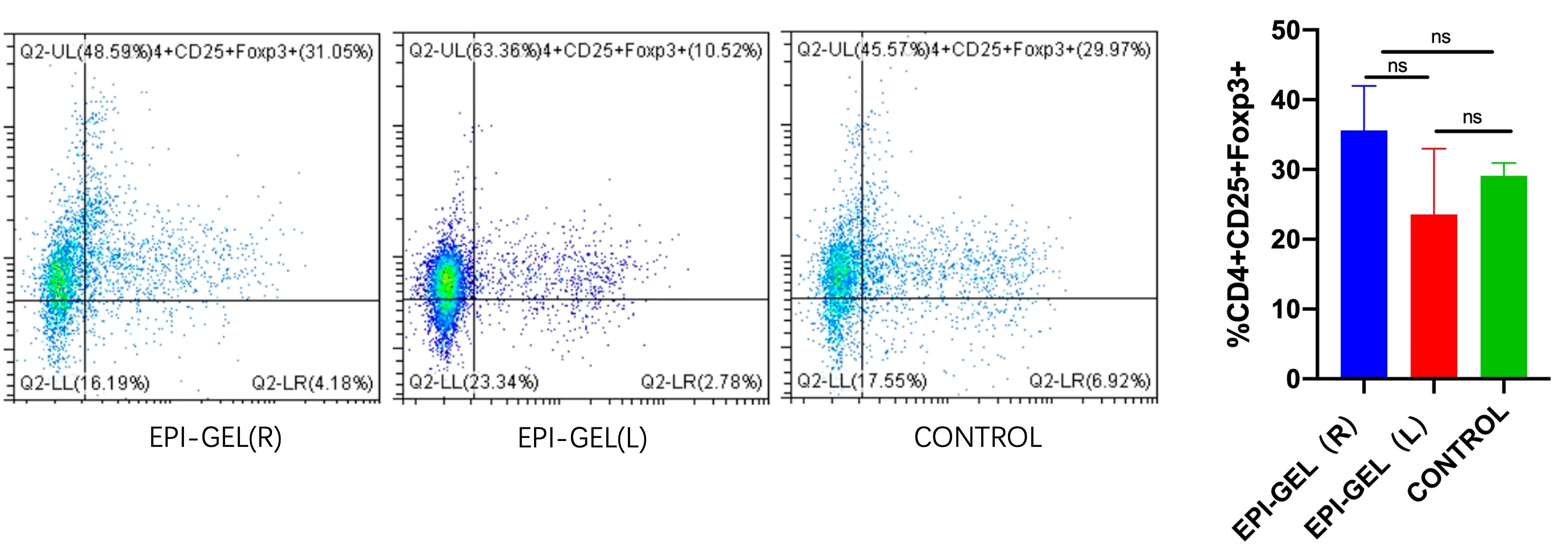

Supplement: Supplementary Figure 1 — Representative flow cytometry analysis of CD4+CD25+Foxp3+ cell infiltration in bilateral tumors of mice treated with epirubicin (EPI) gel. ns, not significant. (one-way ANOVA with Tukey’s test). [file Image_1.jpeg]

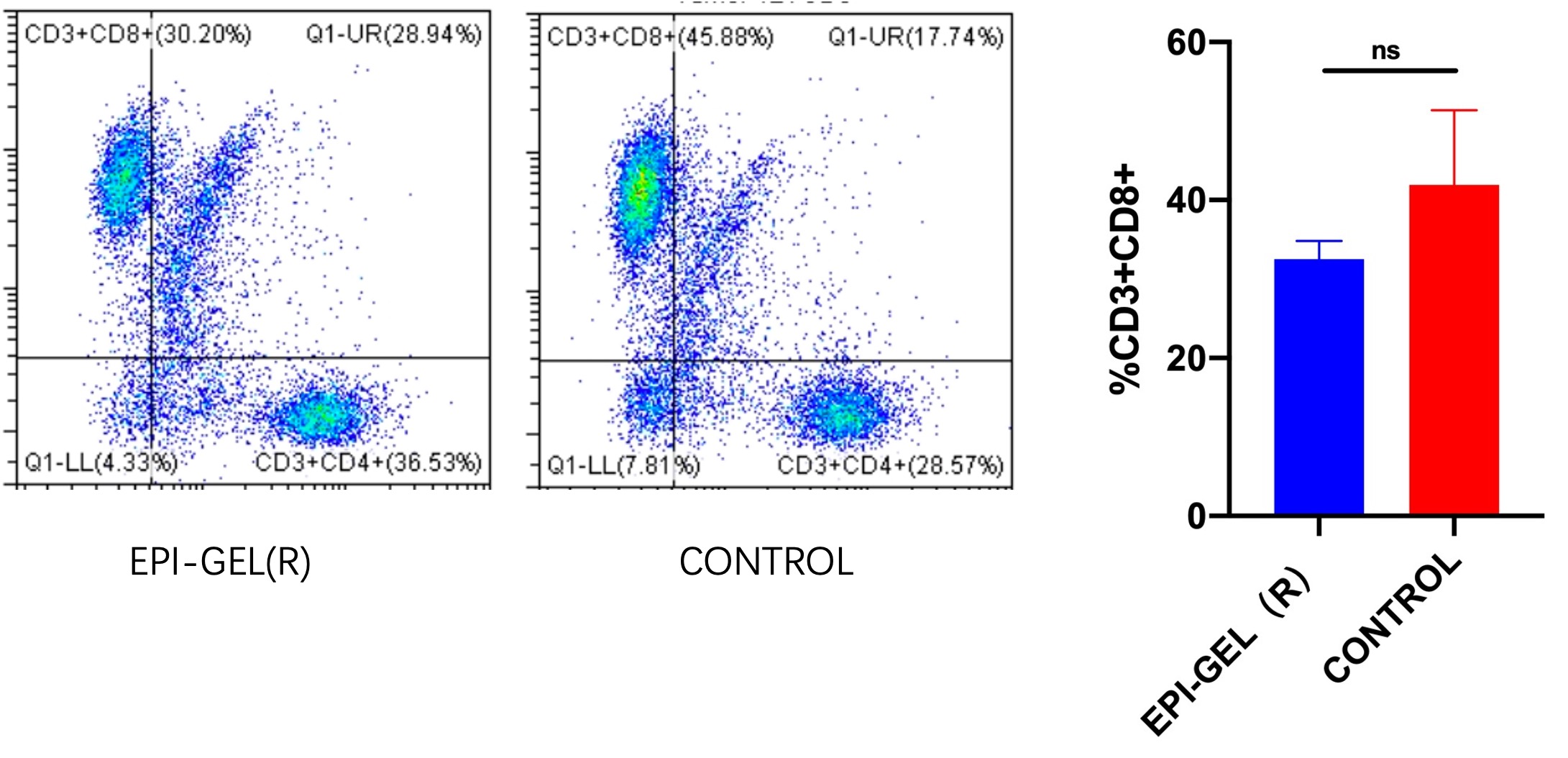

Supplement: Supplementary Figure 2 — Representative flow cytometry analysis of CD3+CD8+ cell infiltration in the right tumor of mice treated with epirubicin (EPI) gel. ns, not significant. (Unpaired t test). [file Image_2.jpeg]
